# Intraoperative open-chest epicardial high-density mapping–guided hybrid ablation for refractory premature ventricular complexes and ventricular tachycardia in a pediatric patient undergoing aortic valve repair

**DOI:** 10.1016/j.hrcr.2026.04.015

**Published:** 2026-04-17

**Authors:** Anjan S. Batra, Roderick Yang, Aaron McKinley, Dan LaBahn, Ashley Hart, Daniel Nento

**Affiliations:** 1 Children’s Hospital of Orange County, Orange, California; 2 University of California, Irvine, Irvine, California; 3 Rainbow Children’s Hospital–Case Western Reserve University, Cleveland, Ohio; 4 Abbott Medical, Abbott Park, Illinois

**Keywords:** Pediatric electrophysiology, Ventricular tachycardia, Premature ventricular complexes, Epicardial mapping, Intraoperative ablation, Structural heart disease, Aortic regurgitation, High-density mapping

**Key Teaching Points**

• Pediatric ventricular arrhythmias may be refractory to medical therapy and standard catheter ablation.
• Intraoperative open-chest epicardial high-density mapping may provide additional anatomic information to help guide ablation in anatomically complex regions.
• A hybrid surgical–electrophysiological approach may be considered during planned cardiac surgery when conventional strategies are limited.

## Introduction

Ventricular arrhythmias in pediatric patients with structural heart disease may be challenging to manage, particularly when refractory to medical therapy and conventional catheter ablation. In selected cases, intraoperative epicardial mapping performed during planned cardiac surgery may facilitate localization of arrhythmogenic substrates that may be incompletely characterized using standard endocardial techniques. We report a case of intraoperative open-chest epicardial high-density mapping used to guide a surgically facilitated hybrid ablation strategy in a pediatric patient with refractory premature ventricular complexes (PVCs) and ventricular tachycardia (VT) undergoing aortic valve repair.

## Case report

**Figure 1:**
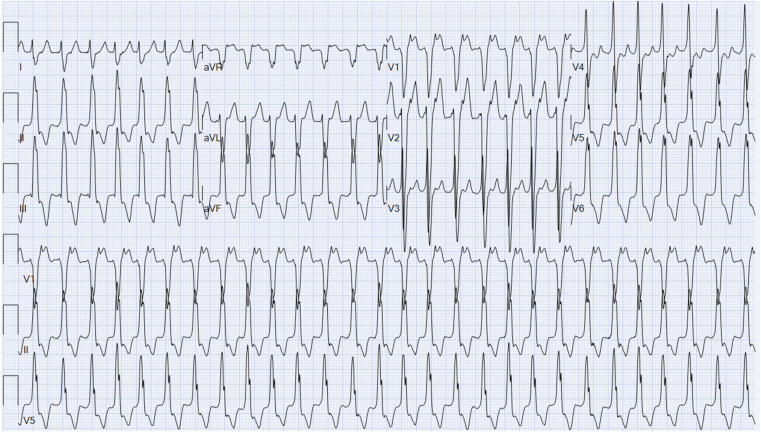
The clinical electrocardiogram during ventricular tachycardia.

A 10-year-old boy presented with frequent nonsustained VT (Figure 1), a high PVC burden (>40%), and progressive left ventricular systolic dysfunction consistent with PVC-induced cardiomyopathy. Medical therapy with nadolol and flecainide resulted in minimal symptomatic or arrhythmic improvement.

An electrophysiology study with endocardial mapping and catheter ablation was performed. Earliest activation was identified in the right ventricular (RV) outflow tract and within the right coronary sinus of the aortic root, and radiofrequency lesions were delivered at these sites. Although transient suppression of ventricular ectopy was achieved, ventricular ectopy recurred shortly after emergence from anesthesia.

After this procedure, the patient developed progressive aortic valve injury resulting in moderate-to-severe aortic regurgitation. Intraoperative inspection confirmed leaflet perforation of the aortic valve, consistent with thermal injury related to previous ablation within the aortic root.

Although repeat endocardial ablation could have been considered, the previous procedure failed to achieve durable arrhythmia suppression and resulted in significant aortic valve injury with leaflet perforation. Given the failure of conventional transvenous catheter ablation and the need for open-heart surgery, a multidisciplinary decision was made to perform intraoperative open-chest epicardial mapping to guide a hybrid ablation strategy at the time of aortic valve repair.

### Intraoperative epicardial mapping

Under general anesthesia, the patient underwent median sternotomy. The pericardium was opened and marsupialized. Targeted surgical dissection was performed between the ascending aorta and pulmonary artery to allow catheter access and stable epicardial contact along the RV infundibulum and conal septal region—an area not accessible via percutaneous epicardial techniques. This dissection was limited and required approximately 10 minutes.

Surface patches for impedance-based electroanatomic mapping were positioned to avoid interference with the surgical field. Warm lactated Ringer’s solution was instilled into the pericardial space to improve electrogram quality and catheter localization. At baseline, the patient exhibited ventricular bigeminy.

**Figure 2:**
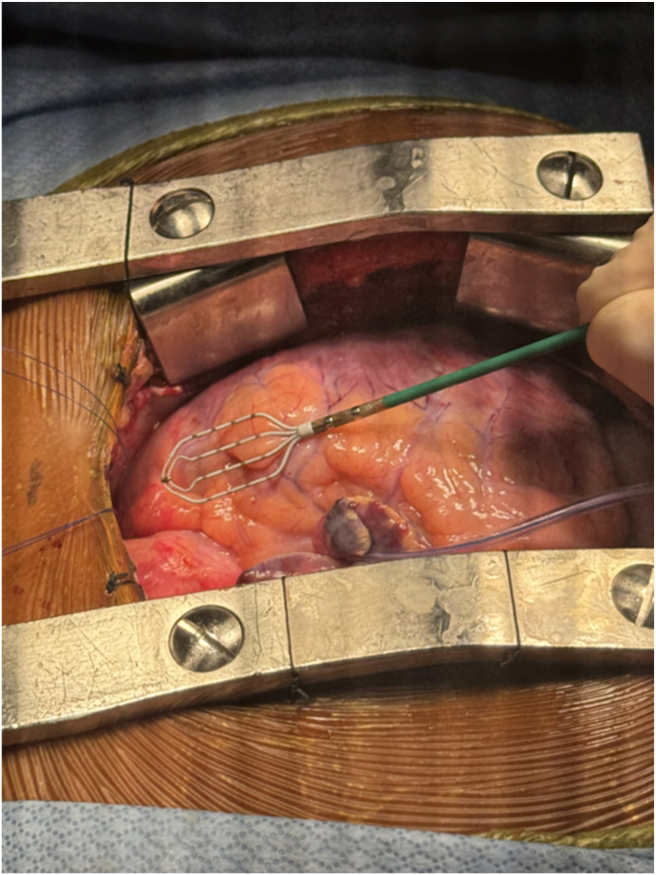
High-density electroanatomic maps were created using the Advisor HD Grid mapping catheter.

**Figure 3:**
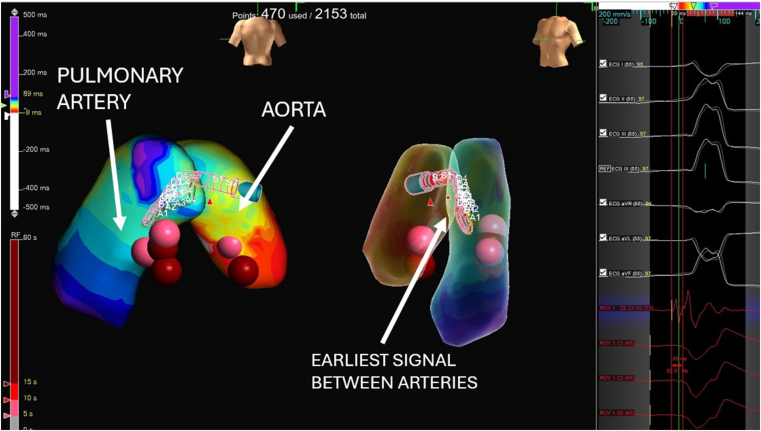
Area with earliest activation localized to the right ventricular infundibulum, just inferior to the pulmonic valve and superior to the aortic valve. *Red dots* indicate the ablation points.

High-density epicardial activation mapping demonstrated earliest activation along the posterior RV infundibulum and conal septal region, inferior to the pulmonary valve and adjacent to the aortic root (Figures 2 and 3). At the site of earliest activation, signals preceded QRS onset by approximately 25–30 ms. Bipolar electrograms were low amplitude and mildly fractionated. Unipolar electrograms demonstrated an initial negative deflection but lacked a sharp QS morphology. PVC morphology demonstrated >95% match at this site. These findings helped localize the arrhythmogenic region and informed the subsequent ablation strategy.

### Ablation strategy

After completion of epicardial mapping, the patient was placed on cardiopulmonary bypass and cardioplegic arrest was achieved. Ablation was performed using a surgically facilitated hybrid approach, guided by epicardial activation timing, previous endocardial findings, and direct intraoperative anatomic visualization.

Although repeat intraoperative endocardial mapping could have provided additional confirmation, the concordance between previous endocardial findings and epicardial activation, along with the need to minimize cardiopulmonary bypass and cross-clamp time, supported proceeding directly to ablation.

Cryoablation was delivered via the tricuspid valve to the posterior, posterolateral, and posteroseptal aspects of the pulmonary valve annulus, with emphasis on the site corresponding to the earliest epicardial activation along the conal septum. Cryoenergy was selected in this region to reduce the risk of injury to adjacent conduction tissue.

The ascending aorta was then opened to allow aortic valve repair. Under direct visualization, radiofrequency ablation was delivered at the right coronary sinus and through the aortic valve onto the conal septum of the left ventricular outflow tract. Impedance and contact force were carefully monitored. Lesion delivery was guided by anatomic landmarks and correlation with previous mapping data.

Because ablation was performed during cardioplegic arrest, immediate suppression of ectopy could not be assessed intraoperatively. However, complete elimination of ventricular ectopy was observed upon return to sinus rhythm postoperatively.

Formal repeat high-density endocardial mapping was not performed intraoperatively. Lesion placement was guided by epicardial activation timing, correlation with previous endocardial ablation sites, and intraoperative anatomic landmarks rather than empiric delivery alone.

### Outcome

Surgical repair of the aortic valve was successfully performed with no residual regurgitation. The patient was rewarmed and weaned from cardiopulmonary bypass without complication.

Postoperatively, complete suppression of PVCs and VT was observed throughout hospitalization. At the most recent follow-up (2 months after the procedure), ambulatory monitoring demonstrated sustained arrhythmia suppression with recovery of left ventricular systolic function.

## Discussion

Management of ventricular arrhythmias in pediatric patients with structural heart disease remains challenging, particularly when arrhythmias are refractory to medical therapy and conventional catheter ablation.^1^ Anatomic distortion, limited catheter access, and proximity to critical structures can constrain the efficacy and safety of standard endocardial approaches in this population. In addition, smaller chamber size and reduced tolerance for prolonged procedures further complicate electrophysiological interventions in children.

In the present case, the patient exhibited a high burden of PVCs and recurrent VT associated with progressive left ventricular systolic dysfunction, raising concern for PVC-induced cardiomyopathy. Despite antiarrhythmic therapy and previous endocardial catheter ablation targeting the RV outflow tract and aortic root, ventricular ectopy recurred, suggesting an arrhythmogenic region that was incompletely addressed by conventional catheter techniques. Importantly, the previous ablation was complicated by progressive aortic valve injury resulting in moderate-to-severe aortic regurgitation, necessitating surgical repair. This circumstance provided a unique opportunity to pursue a combined surgical and electrophysiological strategy.

Epicardial mapping has emerged as an important adjunct in the evaluation of ventricular arrhythmias when endocardial approaches fail or when arrhythmogenic substrates are suspected to reside outside the endocardial surface. Percutaneous epicardial access via subxiphoid puncture is well described in adult populations^2^; however, its applicability can be limited by previous procedures, pericardial adhesions, or proximity to coronary arteries and the phrenic nerve.^3^ In such scenarios, particularly in pediatric patients, direct surgical access to the epicardial space may enable more comprehensive mapping with improved catheter stability and contact.

In this case, intraoperative open-chest epicardial high-density mapping served a primarily diagnostic role, allowing precise localization of earliest activation along the posterior RV infundibulum and conal septal region—an anatomically complex area inaccessible to percutaneous epicardial techniques. These findings provided additional anatomic information that helped guide the ablation strategy. Although epicardial mapping guided the intervention, ablation energy delivery was performed using a surgically facilitated hybrid approach, incorporating both cryoablation and direct endocardial radiofrequency ablation under open surgical visualization.

The ability to perform ablation under direct visualization provided improved anatomic orientation and facilitated controlled lesion delivery in proximity to critical structures, although it does not allow precise localization of the conduction system and must be interpreted in conjunction with electrophysiological data. This approach allowed lesion delivery in anatomically complex regions using both cryoablation and radiofrequency energy, tailored to the intraoperative setting. The use of warm lactated Ringer’s solution in the pericardial space represents a practical procedural adaptation that may enhance mapping fidelity in the open-chest setting by improving electrogram quality and stabilizing impedance-based catheter localization. Such technical considerations are important when translating high-density mapping technologies from closed, percutaneous environments into the operative field.

Although this case demonstrates the feasibility and short-term efficacy of intraoperative epicardial mapping–guided hybrid ablation, several limitations warrant discussion. This is a single case with limited follow-up, and long-term arrhythmia outcomes and lesion durability remain unknown. Formal repeat high-density endocardial mapping was not performed intraoperatively, and termination strips and detailed intraoperative electrogram recordings were not available, limiting precise electrophysiological correlation at ablation sites. In addition, this strategy requires close multidisciplinary collaboration, specialized equipment, and institutional expertise, which may limit broader applicability. Further experience and systematic study are needed to better define patient selection, procedural risks, and long-term outcomes for intraoperative epicardial mapping–guided ablation strategies in pediatric populations.

## Conclusion

Intraoperative open-chest epicardial high-density mapping can be used to guide hybrid ablation strategies in pediatric patients with refractory ventricular arrhythmias undergoing planned cardiac surgery. Although ablation energy delivery may be endocardial, epicardial mapping may provide additional anatomic information to guide ablation that may not be achievable with conventional catheter-based approaches alone.

## Disclosures

Mr McKinley and Mr LaBahn are employees of Abbott Medical. The other authors have no conflicts of interest to disclose.
